# Why does Existential Threat Promote Intergroup Violence? Examining the Role of Retributive Justice and Cost-Benefit Utility Motivations

**DOI:** 10.3389/fpsyg.2015.01761

**Published:** 2015-11-20

**Authors:** Gilad Hirschberger, Tom Pyszczynski, Tsachi Ein-Dor

**Affiliations:** ^1^Baruch Ivcher School of Psychology, Interdisciplinary Center HerzliyaHerzliya, Israel; ^2^Psychology Department, University of Colorado at Colorado SpringsColorado Springs, CO, USA

**Keywords:** terror management, intergroup conflict, justice beliefs, cost-benefit analysis, support for violence

## Abstract

The current research examined the role of retributive justice and cost-benefit utility motivations in the process through which mortality salience increases support for violent responses to intergroup conflict. Specifically, previous research has shown that mortality salience often encourages political violence, especially when perceptions of retributive justice are activated. The current research examined whether mortality salience directly activates a justice mindset over a cost-benefit utility mindset, and whether this justice mindset is associated with support for political violence. In Study 1 (*N* = 209), mortality salience was manipulated among Israeli participants who then read about a Hamas attack on Israel with either no casualties or many casualties, after which justice and utility motivations for retribution were assessed. Study 2 (*N* = 112), examined whether the link between death primes and support for an Israeli preemptive strike on Iran’s nuclear facilities is mediated by justice or cost-benefit utility considerations. Results of both studies revealed that primes of death increased justice-related motivations, and these motives, rather than utility motives, were associated with support for violence. Findings suggest that existential concerns often fuel violent intergroup conflict because they increase desire for retributive justice, rather than increase belief that violence is an effective strategy. These findings expand our knowledge on the motivations for intergroup violence, and shed experimental light on real-life eruptions of violent conflict indicating that when existential concerns are salient, as they often are during violent conflict, the decision to engage in violence often disregards the utility of violence, and leads to the preference for violent solutions to political problems – even when these solutions make little practical sense.

## Introduction

“*My administration has a job to do and we’re going to do it. We will rid the world of the evil-doers*.”

President George W. Bush – 9/16/01

“*The paradox is that evil comes from man’s urge to heroic victory over evil*.”

Becker (1975, p. 136)

Prominent social thinkers such as Fromm (1941), Rank (1958), Becker (1975), and Lifton (2003) have all noted that the pursuit of goodness, justice, and a better world has been paradoxically responsible for some of humankind’s most horrendous acts of violence. The stated goal of the Nazi genocide of European Jews was to promote a superior race of humans, and to purify the world by eliminating those they viewed as inferior. Osama Bin Laden described his motivation for the 9/11 terrorist attacks on the World Trade Center and Pentagon as providing retribution for injustices in the Middle East ostensibly committed by the US. Interviews with rank-and-file terrorists reveal that feeling that one’s people have been unjustly treated is among the most frequently reported motivators for their attacks (e.g., Stern, 2003; Richardson, 2006).

These observations regarding the paradoxical relationship between justice and violence are substantiated by research showing that the belief that violence is a necessary means to a moral end is powerful enough to trump rational cost benefit considerations regarding the utility of violence. For example, research on Americans, Nigerians, and Israeli West-Bank settlers show that support for military interventions reflects deontological reasoning focused on what is morally appropriate or just, rather than on what is practically useful (Ginges and Atran, 2011). Similarly, perceptions of defeat in a previous violent encounter compared with perceptions of victory increased support for violent retaliation, even though defeat suggests that violence is counter-productive (Ein-Dor and Hirschberger, 2013). The research reported in this paper assessed the possibility that at least part of the motivation underlying support for political violence to restore justice is the protection from existential anxiety that retribution for perceived injustices provides. Specifically, we contend that the link observed in numerous studies between existential concerns and political violence (see Hirschberger and Pyszczynski, 2011 for a review) may reflect the effects of existential concerns on justice motivation, and that this justice motivation plays an important role in promoting intergroup violence. Thus, existential concerns often increase the motivation to pursue justice, and this need for justice often leads to violent behavior aimed at vanquishing an evil enemy.

### The Paradox of Justice and Violence

To suggest that intergroup aggression is motivated by justice concerns may appear counter-intuitive, at first sight, because justice connotes fairness, morality, virtues, and ethics that should ostensibly promote better intergroup understanding. The intuitive alternative to our proposal is that conflict is actually driven by naked self-interest that pays no heed to justice and morality. Although the concrete consequences of specific incursions by an adversary surely play a role in promoting support for violence (Bueno de Mesquita, 1988), we argue that it is the symbolic implications of these actions that are most important in motivating people to support war and other forms of political violence. The flourishing research on justice and morality suggest a *homo moralis* model of human motivation (Skitka, 2009), wherein the need to perceive our behavior within a moral framework often overcomes economic cost-benefit considerations.

In the context of intergroup relations, research has demonstrated that aggression rarely, if ever occurs without justification, and that in order to harm another person or group, justifying mechanisms such as dehumanization, moral disengagement, and exclusion from the moral community must be activated (Bandura et al., 2001; Castano and Giner-Sorolla, 2006). We propose that existential concerns constitute a powerful underlying motivation for both justice-related and violent motivations, and employ terror management theory (TMT: Greenberg et al., 1997; Pyszczynski et al., 2015) as an existential framework that can illuminate how the desire to promote justice and morality may paradoxically, and tragically, fan the flames of conflict.

### Existential Roots of Justice and Violence

Terror management theory posits that people are protected from the potential for anxiety that results from their awareness of the inevitability of death by an anxiety buffering system consisting of: (1) their cultural worldviews, which provide an explanation for existence, standards through which they can attain a sense of personal value, and the promise of literal or symbolic immortality to those who live up to these standards, (2) self-esteem, which is acquired by believing in the cultural worldview and living up to its standards, (3) and close interpersonal attachments that validate one’s worldview and self-esteem. Because these psychological entities play a vital role in protecting people from deeply rooted fears, much social thought and behavior is oriented toward maintaining and defending them against threats (For recent reviews of evidence for the fundamental propositions of TMT, see Kesebir and Pyszczynski (2012), Greenberg et al. (2014), or Pyszczynski et al., 2015).

Because consensual validation of worldviews and self-conceptions is needed for effective protection against anxiety, the mere existence of those with different worldviews is threatening. Divergent worldviews become even more threatening when members of other groups threaten one’s collective self-esteem by proclaiming the superiority of their worldview. Such proclamations undermine the conviction that one’s group represents absolute values of truth and goodness, and therefore increase anger, derogation, and aggression against any source that contests the moral supremacy of one’s group (Eidelson and Eidelson, 2003). Research has shown that such reactions to challenges to one’s group are especially powerful when thoughts of death are salient (Pyszczynski et al., 2008).

In addition to the threat to collective self-esteem posed by harm to one’s group and the threat to cultural worldviews posed by disagreement with core group beliefs and values, a major consequence of mistreatment by an adversary is that such actions violate the principles of justice and fairness that provides security in a dangerous world. As Lerner (1980) pointed out, people are strongly motivated to view the world as just because this implies that one can avoid negative outcomes by being a good person.

From a TMT perspective, justice is especially important for the regulation of existential concerns. Assuming that the world is just provides order, structure, and emotional security by implying that behavior is related to outcomes. Self-esteem can provide security only if one assumes the world is just – only in a just world does being a good person guarantee good outcomes. Thus, assumptions about justice are necessary for self-esteem (whether individual or collective) to serve its anxiety buffering function. The assumption that the world is just also implies that bad behavior or affronts to oneself or one’s group must be punished. The absence of such retribution would imply that either the world is *not* just or that one (or one’s group) lacks value and is deserving of negative treatment.

The motivation to pursue justice, therefore, reflects an existential necessity because construing the world as a just place in which the good are rewarded and the evil are punished provides comfort and shields people from the possibility of a capricious, random universe wherein happenstance rules and evil may triumph. Indeed, research has shown that mortality salience increases justice motivations, as evidenced by greater derogation of victims and preference for stories in which tragedy leads to positive outcomes (e.g., Landau et al., 2004; Hirschberger, 2006). Moreover, exposure to instances of injustice increases the accessibility of death related thoughts (Hirschberger, 2006).

The effects of mortality salience on justice strivings may sometimes have prosocial implications, such as increasing sensitivity to injustices (Kastenmüller et al., 2013). But more often than not, the effects of existential concerns on justice are reflected in the desire to rationalize and justify human suffering (Hirschberger, 2015), and more severely punish social transgressors (Florian and Mikulincer, 1997). Thus, we suggest that the effects of mortality salience on justice perceptions in the context of intergroup conflict are often driven by the desire to justify violence – a tendency that is typically seen as violating norms of justice and morality.

A large body of research indicates that mortality salience manipulations often increase support for political violence in response to pressing real world conflicts. Specifically, mortality salience increased support for American military actions in Iraq (Landau et al., 2004); led Israelis to support a pre-emptive strike against Iran and against the Hezbollah in Lebanon (Hirschberger et al., 2009); led conservative Americans to support extreme military tactics to fight terrorism (Pyszczynski et al., 2006); led right-wing Israelis to support violent resistance against policies that threaten their worldview (Hirschberger and Ein-Dor, 2006); and led Iranians to increase their support for suicide bombing as a tactic to fight American imperialism (Pyszczynski et al., 2006). TMT researchers have interpreted these findings as suggesting that existential threat increases the tendency to construe violence as a justifiable and heroic response to an evil adversary (e.g., Pyszczynski et al., 2006).

In fact, many of the conditions under which MS increases support for political violence involve construing violence as moral, just and as reflecting the values of one’s worldview. In some studies, this was an explicit induction, as when Israelis read a report of a speech from Iranian leaders calling for the destruction of Israel (Hirschberger et al., 2009); such proclamations are likely to be perceived as justifying military aggression, and, indeed, MS increased support for a preemptive strike against Iran when participants were exposed to such rhetoric, but not when they were exposed to more conciliatory remarks. In assessments of the psychological processes involved in Americans’ reactions to the 9/11 terrorist attacks (e.g., Landau et al., 2004; Pyszczynski et al., 2006), participants reminded of these attacks were likely to view violence as justified even in the absence of direct manipulations of such construals. Approximately two months following the attacks, Kaiser et al. (2004) found that the more participants endorsed just world beliefs before the attacks, the more they desired revenge.

### The Present Research

Although these findings can be interpreted as indicating an increased desire to vanquish an evil enemy and restore justice (Pyszczynski et al., 2006), the role of justice concerns in these effects has only recently been directly assessed. In research conducted on Israeli Jews, Arabs, and South Koreans, primes that emphasized the importance of justice increased support for political violence when mortality was salient, even when the practical utility of violence was explicitly stated to be low (Hirschberger et al., in press). Specifically, this research showed that Palestinian citizens of Israel who were guided to think about the justice of the Palestinian side in the conflict with Israel responded to MS with greater support for violence against Israel. When asked to think about the possible costs and benefits of escalating the conflict with Israel, however, MS had the opposite effect and reduced support for violence. Similarly, South Koreans and Israeli Jews who were exposed to descriptions of violent attacks against their people (high justice condition) responded to MS with increased support for retaliation even if they were exposed to military experts suggesting that a counter-attack would have no expected utility (low utility condition). These studies demonstrated the interaction between existential concerns and justice and utility considerations, but they did not address a more fundamental question – do existential concerns actively increase the appeal of justice considerations over cost-benefit utility ones?

The current research builds on previous findings showing that deontological reasoning takes precedence over utilitarian reasoning (Ginges and Atran, 2011), especially when death is salient (Hirschberger et al., in press). But the present studies go beyond previous findings to test the hypothesis that retributive justice motivation is increased by mortality salience, and that the link between such threat and the desire to aggress against an adversary is driven by retributive justice rather than cost-benefit utility concerns.

## Study 1

Study 1 examined whether MS makes participants more supportive of arguments for political violence based on the rhetoric of justice over arguments based on the cost-benefit utility of violence. To better understand the effects of MS on retributive justice, we also examined whether MS increases support for violence primarily when the instigation to restore justice is strong. Darley and Pittman’s (2003) analysis of retributive justice posits that the greater the harm the other party inflicts, the greater the desire for retaliation. Conversely, when little harm has been done, the motivation for retribution is likely to be low. If MS increased support for retributive attacks primarily in response to severe incursions by an enemy when the desire for justice is therefore high, it would provide support for our claim that concerns for justice play an important role in the relationship between existential threat and support for political violence. We, therefore, created two conditions, one in which retaliation was highly justified by the severe consequences of an attack, and another in which it would be difficult to justify a violent reprisal because the consequences of the instigating attack were minimal. Participants were primed with either MS or an aversive control topic before responding to these scenarios.

To address these issues within the context of the Israeli–Palestinian conflict, we constructed a measure of justice and utility motivations for violence (the justice-utility scale, JUS) that provides distinct assessments of justice and utilitarian motivations for violence, Study 1 was designed to determine whether MS increases support for justice, utility, or both motivations, and whether the effect of MS emerges primarily in response to severe provocations, when the desire for justice is likely to be greater. Thus, in the current study participants read one of two scenarios about a missile attack on the town of Sderot (which is located only one mile from the city of Gaza); half of the participants were randomly assigned to a scenario depicting a severe outcome of the missile attack with many Israeli casualties, and the other half to a scenario depicting a mild outcome with no casualties.

### Method

#### Participants

Two hundred and nine undergraduate students, 57 men and 151 women (one participant did not report gender), ranging in age from 19 to 55 (*Median* = 24, SD = 4.2), participated in the study for course credit. The sample size was determined by a power analysis (Faul et al., 2007) to allow 80% power for detecting weak-sized within-between interaction (the main analysis in the current study; 1% explained variance). The analysis indicated that we need at least 198 participants. The observed power in Study 1 (with 209 participants) is, therefore, 82.44%. Both studies reported here were approved by the institutional review board (IRB) of the IDC.

#### Materials and Procedure

Experimental sessions were run in groups of 10–15 people with participants randomly assigned to experimental conditions within each session. Participants were first randomly assigned to either the *mortality salience* or *pain salience* conditions. In the *mortality salience condition*, participants answered the following open-ended questions: “What do you think happens to you as you physically die and once you are physically dead?” and “Please briefly describe the emotions that the thought of your own death arouses in you.” In the *pain salience condition*, participants received the same open-ended questions with references to death replaced with “severe physical pain.” Research has repeatedly shown the effectiveness of this procedure to increase thoughts of death (e.g., Pyszczynski et al., 2006). Then, all participants completed a word search puzzle that served as a distraction. Following this procedure, all participants read a description of a missile attack from the Gaza Strip on the Israeli town of Sderot. In half the cases, the attack was described as a lethal attack with many casualties. In the other half, the attack was described as mild with damage to buildings but no human casualties.

Following these scenarios, participants completed the JUS, a 26-item measure answered on 7-point scales ranging from *strongly disagree* (1) to *strongly agree* (7). The JUS includes 10 retributive justice items (e.g., “A military strike on Gaza will make the Palestinians pay for their crimes”; “We should attack Gaza because what they are doing to us is unjust”; “attacking Gaza will do justice for Israelis who were killed by Hamas”; “it would be completely unjust to attack the Gaza strip (*R*)”; “An attack on Gaza would avenge the death of Israelis”), six utility items (e.g., “A military strike on Gaza will effectively reduce missile attacks against Sderot”; “An attack on Gaza will restore our deterrence”; “An attack on Gaza will weaken Hamas”; “An attack on Gaza will serve no purpose (R)”; “Killing the leaders of Hamas will deliver a powerful warning to Israel’s enemies”), and 10 filler items (e.g., “The government should subsidize public transportation”; “I care about the environment”; “the interest rate is too high”; “the government’s health care plan should be expanded”; “there should be government control over the supreme court”). To examine the construct validity of the JUS, we administered the scale to an independent sample of 205 undergraduate participants (48 men, 154 women; 3 participants did not report gender) who completed the scale for course credit. A maximum likelihood-based factor analysis with Oblimin rotation conducted on the JUS revealed intact structural validity and supported the two-factor solution, with the justice factor explaining 35.4% of the variance (*M* = 4.06, *SD* = 1.65; with factor loading ranging from 0.99 to 0.63*)* and the utility factor adding 17.95% to the explained variance *(M* = 4.96*, SD* = 1.24; with factor loading ranging from 0.86 to 0.62). Cronbach’s alphas were adequate (0.94 and 0.74 for the justice and utility subscales, respectively), and scores were computed for each participant by averaging the relevant items. Next, participants answered a demographic questionnaire and were debriefed.

### Results and Discussion

To examine the effects of mortality salience (death, pain) and threat severity (severe, mild) on the two JUS factors (justice, utility), we conducted a mixed-designed analysis of variance, in which the between-subject independent factors were mortality salience and threat severity, and the within-subject independent factor was the JUS dimension (justice and utility). Preliminary Pearson correlation analyses indicated that political orientation was associated with the JUS factors such that right-wing views were associated with greater support for justice-motivated retributions, *r*_(207)_ = 0.36, *p* < 0.001, and less support for utility-motivated retributions, *r*_(207)_ = –0.14, *p* = 0.047. We, therefore, added political orientation as a covariate to adjust the analysis for its contribution (the pattern of results remain the same when not controlling for political orientation; the slopes were homogenous, *F*_(2,199)_ = 0.49, *p* = 0.62 for justice-motivated retributions, and *F*_(2,199)_ = 1.86, *p* = 0.16 for utility-motivated retributions, and thus the basic assumptions of ANCOVA were met).

**FIGURE 1:**
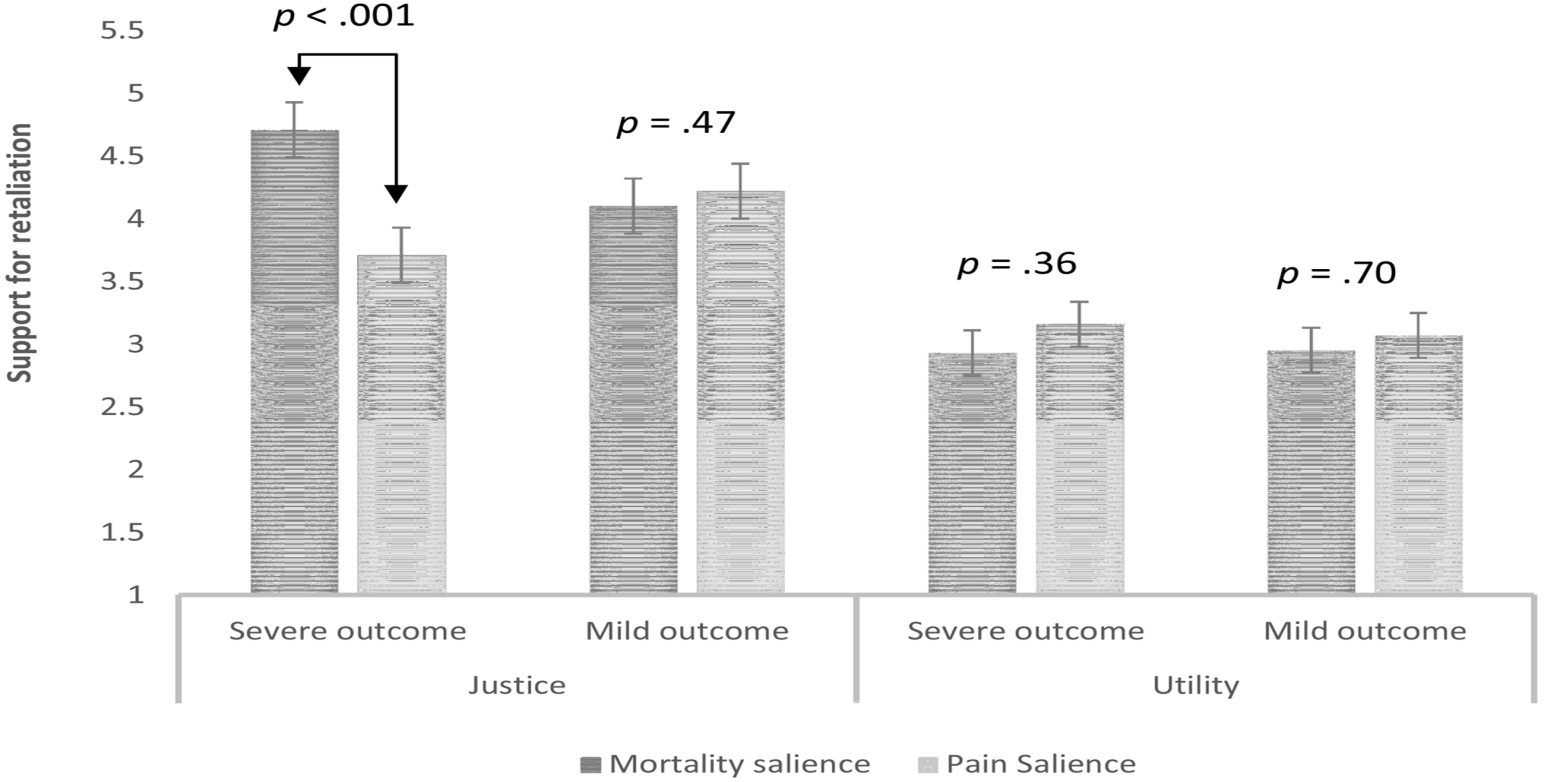
**Mortality salience increases support for violent retaliations for justice- and not utility-related reasons when justification for a retaliation is high**.

The analysis revealed the expected 3-way interaction between mortality salience, threat severity and type of JUS dimension, *F*_(1,200)_ = 4.73, *p* = 0.003, $\eta_{p}^{2}$ = 0.02. Simple effects test with Šidák (1967) adjustment indicated that the simple 2-way interaction between mortality salience and threat severity was significant for justice-motivated retributions, *F*_(1,200)_ = 9.38, *p* = 0.002, $\eta_{p}^{2}$ = 0.05, but not for utility-motivated retributions, *F*_(1,200)_ = 0.15, *p* = 0.70, $\eta_{p}^{2}$ = 0.00. In line with predictions, mortality salience increased support for justice-motivated retributions when the threat was severe (*p* < 0.001, *Cohen’s d* = 0.67), but not when the threat was mild (*p* = 0.47, *Cohen’s d* = –0.07). Results are summarized in **Figure 1**.

These findings support our hypothesis that MS increases support for justice but not utility-based motivations for violence. Specifically, MS increased the belief that retributions against Hamas would restore justice, but did not increase the belief that such attacks would be pragmatically useful in reducing future attacks. As predicted, this finding emerged only when the initial attack on Israel produced severe outcomes that presumably created a strong desire for retribution. This provides further evidence for the role that justice concerns play in the link between MS and support for political violence. It appears that MS increases support for violent retribution only when there is strong provocation that leads violent retribution to be viewed as just, when people are perhaps experiencing what Darley and Pittman (2003) referred to as “moral outrage.” Indeed, all previous studies showing that MS increased support for political violence were conducted within the context of long-standing political conflicts in which there were many instances of egregious actions on the part of the adversary that likely led participants to believe that violence was a just response. The present findings document the role of perceived justification is these types of effects; they also suggest, however, that when the outcome of an adversary’s attack is relatively inconsequential, cooler heads seem to prevail.

## Study 2

Study 1 demonstrated that MS increases the motivation to restore justice, but does not significantly influence utility-based motives for violence; this occurred only when the transgression leading up to the decision regarding a retaliative attack produced relatively severe consequences. This is consistent with Darley and Pittman’s (2003) proposition that the motivation to retaliate is directly proportional to the harm brought on by the instigating event. These findings also suggest that an increased desire for justice may mediate the oft-found effect of MS on support for political violence against an adversary.

In Study 2, we went a step further and examined whether there is a causal chain wherein MS increases justice-related cognitions in general, rather than those specifically focused on the conflict at hand, which then increase support for violent solutions to conflict. We also wished to extend our conclusions beyond Israel’s conflict with the Palestinians. Thus, in Study 2, our dependent measure was focused on Israeli participants’ support for a preemptive strike on Iran’s nuclear facilities. Many Israelis view Iran as the most imminent threat to their existence (Ben Meir and Shaked, 2007), but opinion polls indicate that they feel ambivalent about initiating a military campaign against Iran (Telhemi, 2012). Based on our previous research, which suggests that MS does not induce violent reactions against Iran when violence is perceived as avoidable or costly (Hirschberger et al., 2009), we predicted that the effects of MS on support for a preemptive strike on Iran would lead to some ambivalence and hesitation. Thus, we hypothesized that MS would induce support for preemptive violence only when thinking in justice-related terms, and that in other cases there will either be no significant association between MS and support for preemptive violence, or MS may even reduce the proclivity for violence. Addressing these effects within the context of the debate regarding the possibility of Iran developing a nuclear weapon seems especially telling because discussion of military action to prevent this from occurring is typically focused on the utility rather than justice of such actions.

### Method

#### Participants

Israeli undergraduate students (*N* = 112), 25 men and 87 women, aged 20 to 36 (*Median* = 23, *SD* = 2.2), participated in the study for course credit. Participants were recruited through the psychology subject pool at the IDC and were directed to the Qualtrics system on which the experiment was conducted. The sample size was determined by Fritz and MacKinnon’s (2007) power analysis simulation to allow 80% power for detecting moderated-sized mediation paths (the main analysis in the current study). The simulation indicated that we need at least 115 participants. We fell slightly short of this recommendation because data collection terminated at the end of the semester.

#### Materials and Procedure

Participants registered for a Qualtrics-based online experiment. Following a consent form, participants completed a filler personality inventory, were randomly assigned to either the MS or control condition as in Study 1, and answered 10 questions about their daily habits that were included as a delay and distraction.

Next, participants completed a 9-item questionnaire that assessed their general tendency to seek justice, independent of the specific context (four items; “When somebody hurts you, you want to get back at them”; “You should fight for a just cause, even if other people find it pointless”; “Your feeling of justice is very important to you”; “You don’t insist on being right if there is no point in it (*R*)”; α = 0.74) and cost-benefit utility (five items; “You do things to achieve a specific goal, and not because it’s the right thing to do”; “During an argument you try to be rational and practical”; “Your friends know that you would never get into an argument that you couldn’t gain something from”; “You are willing to incur losses for the sake of being right (*R*)”; “You conduct yourself by reason, and not by what feels right”; α = 0.60). Participants rated the extent to which each item described their attitudes on 7-point scales ranging from *strongly disagree* (1) to *strongly agree* (7). A maximum likelihood-based factor analysis with Oblimin rotation revealed adequate structural validity supporting the 2-factor solution, with the justice factor explaining 26.62% of the variance (with factor loading ranging from 0.92 to 0.48) and the utility factor adding 19.92% to the explained variance (with factor loading ranging from 0.55 to 0.36). For each participant, we calculated the tendency to seek justice and to seek cost-benefit utility by averaging the items of each subscale.

Then, participants completed the *aggression toward Iran scale* (Hirschberger et al., 2009), an 11-item measure answered on 7-point scales ranging from *strongly disagree* (1) to *strongly agree* (7), assessing support for a military strike on Iran [e.g., “The IDF should strike Iran’s nuclear facilities”; “Israel should launch a preemptive nuclear attack against Iran”; “The only solution to the crisis with Iran is a diplomatic solution”(*R*)]. We computed a total score by averaging the responses to all items (*M* = 4.3*, SD* = 0.88, α = 0.75). After responding to this measure, participants answered a demographic questionnaire and were debriefed.

### Results and Discussion

To examine whether MS increased participants’ tendency to seek justice, but not cost-benefit utility, we conducted a mixed-design analysis of variance, in which the between-subject independent factors was MS, and the within-subject independent factor was general attitudes about justice and utility. The analysis revealed the expected 2-way interaction between MS and justice/utility, *F*_(1,109)_ = 4.30, *p* = 0.04, $\eta_{p}^{2}$ = 0.04. Simple effects tests with Šidák (1967) adjustment indicated that, in line with predictions, MS significantly increased participants’ tendency to think in justice-related terms (*M* = 4.91, *SD* = 1.02 for the mortality salience condition; *M* = 4.49, *SD* = 1.16 for the control condition; *p* = 0.042, *Cohen’s d* = 0.38), but did not significantly affect utility considerations (*M* = 4.36, *SD* = 0.93 for the mortality salience condition; *M* = 4.47, *SD* = 0.89 for the control condition; *p* = 0.54, *Cohen’s d* = –0.12). We also examined whether there is a direct effect of MS on support for an attack on Iran. An independent samples *t*-test revealed that MS did not directly affect support for an attack on Iran *t*_(109)_ = 0.86, *p* = 0.39, *Cohen’s d* = 0.16.

To establish an indirect link from MS through the tendency to seek justice and/or utility to support for an attack on Iran, we followed three steps based on Preacher and Hayes’ (2008) procedure. First, we examined whether MS influenced participants’ tendency to seek justice and/or cost-benefit utility. Next, we examined whether participants’ seeking of justice and/or cost-benefit utility significantly predicted their level of support for an attack on Iran after controlling for MS. Finally, using accelerated bias-corrected bootstrap analysis, we examined whether the indirect path from MS through the pursuit of justice and/or cost-benefit utility to support for an attack on Iran was significant. As in Study 1, we added political orientation as a covariate to adjust the analysis for its contribution [the pattern of results remains the same when not controlling for political orientation; homogeneity of slopes was met as indicated by the non-significant interactions between political orientation and mediation paths (all *p*s > 0.25)].

**FIGURE 2:**
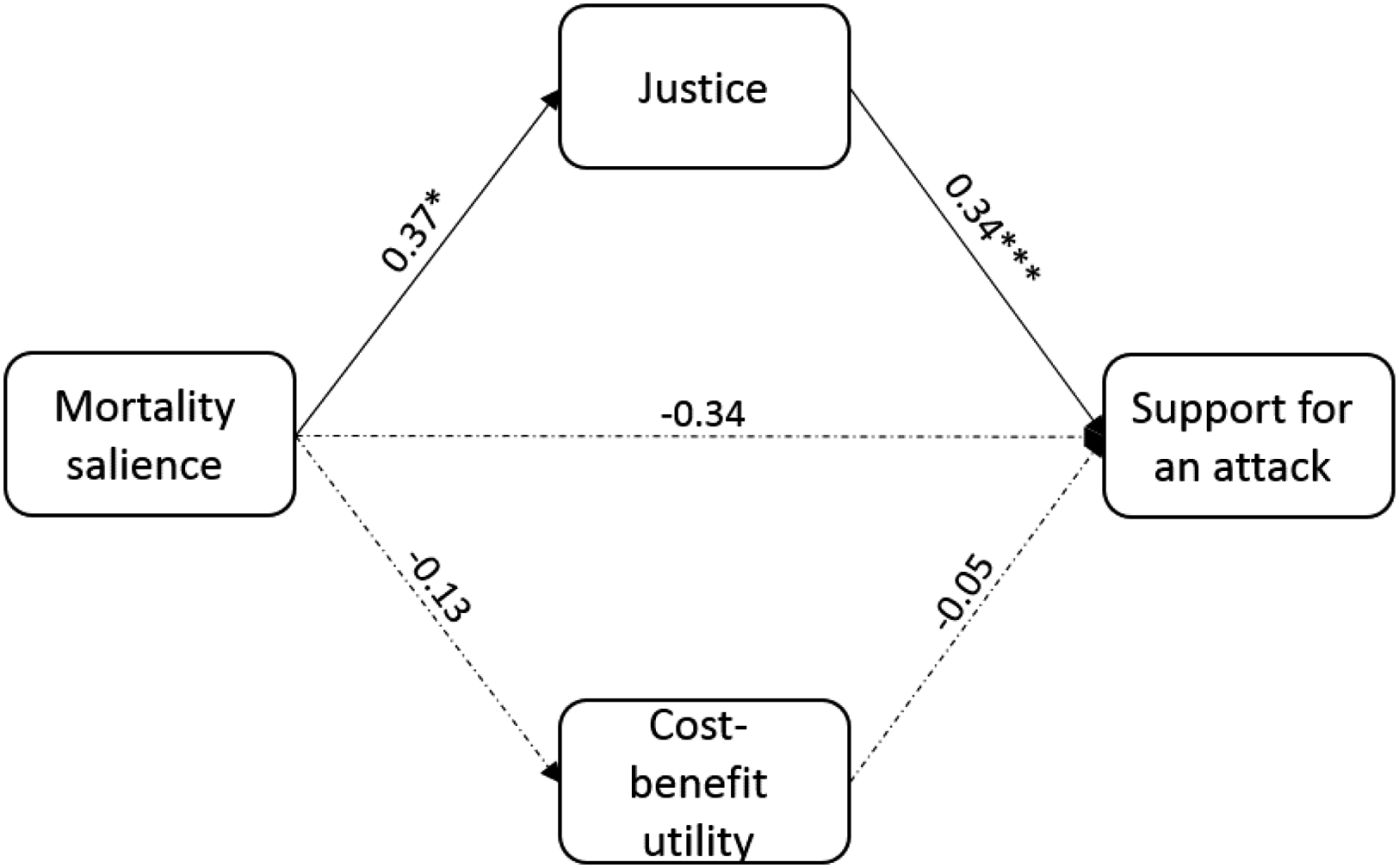
**The effect of mortality salience on support for a preemptive strike on Iran is mediated through justice motivations, but not through cost-benefit utility considerations (coefficients are unstandardized)**. Continuous lines represent significant prediction paths, whereas dashed lines represent non-significant paths.^∗^*p* < 0.05, ^∗∗∗^*p* < 0.001.

The analysis revealed a greater tendency to seek justice (*b* = 0.37, *p* = 0.047; note that *b* refers to unstandardized slopes because standardized slopes are not available in mediation-based analyses) after MS (compared with the control condition), but not a greater tendency to seek cost-benefit utility (*b* = –0.13, *p* = 0.51). The analysis also revealed that controlling for MS, a greater tendency to seek justice (*b* = 0.34, *p* < 0.001) but not a tendency to seek cost-benefit utility (*b* = –0.05, *p* = 0.61), was related to greater support for an attack on Iran. An accelerated bias-corrected bootstrap analysis revealed that the indirect mediating path between MS and support for an attack on Iran via the tendency to seek justice was significant (95% CI: 0.02, 0.31; thus, significantly different from a state of no association), whereas the mediating path via the tendency to seek cost-benefit utility was not significant (95% CI: –0.02, 0.11). The direct link between MS and support for an attack on Iran, however, was not significant (*b* = –0.18, *p* = 0.39, when not controlling for justice and utility, and *b* = –0.34, *p* = 0.10, after controlling for these measures). The model accounted for 25.64% of the variance in support for an attack on Iran (see **Figure 2**).

The findings of Study 2 add to Study 1 by showing that MS induces a general justice mindset but not a utility mindset, and that this justice mindset indirectly increases support for military action. One may wonder why MS did not directly increase support for violence in Study 2. First, in this study, unlike Study 1, participants were asked to support preemptive violence against a formidable foe that many Israelis are reluctant to support military action against, because of the possible devastating consequences (Telhemi, 2012; Ein-Dor and Hirschberger, 2013). Previous research has indicated that when violence is seen as avoidable and when the threat of counter-retaliation is high, MS reduces support for violence (Hirschberger et al., 2009). In the current research there was, indeed, a non-significant trend toward less support for violence in the MS condition. Thus, it seems that MS induced an ambivalent attitude toward violence in this study that is consistent with our previous research on support for violence against Iran (Hirschberger et al., 2009; Ein-Dor and Hirschberger, 2013). Although MS increases the need to uphold the cultural worldview, it also induces a reluctance to act violently for self-protective reasons. The ambivalent effect of MS on violence against a formidable foe (Iran) seems to have suppressed any direct influence of MS on support for violence. When adding justice into the equation, however, an indirect path from MS to justice motivations to support of violence is revealed, providing further support to the main premises of this research that the link between existential threats and justice motivations may promote violence.

## General Discussion

The findings of the two studies reported here converge in showing that existential threat plays an important role in motivating justice-oriented thinking and support for aggressive policies: In Study 1, MS increased the appeal of justice- but not utility-oriented arguments for violent reprisals, and in Study 2, MS increased more general justice, but not utility-related mindsets, which was associated with increased support of military action. Together, these findings suggest that when thinking about possible violent solutions to intergroup conflicts, and when existential threat is salient, as often is the case in violent conflict, people typically rely on justice-based or deontological reasoning, in which considerations of right, wrong, and social identity trump rational cost-benefit analyses. Previous research has already demonstrated that existential concerns often underlie violent intergroup conflict (see Hirschberger and Pyszczynski, 2011 for a review), and that activating concerns over morality and retributive justice override cost-benefit considerations in the context of intergroup conflicts (Ginges and Atran, 2011; Hirschberger et al., in press). These experimental findings are in agreement with a historical analysis of armed conflicts in the past two centuries that concluded that justice motivations were the main impetus for war, and carried more weight in the decision to engage in violence, than rational concerns over security or tangible resources (Welch, 1993). The current research ties together these disparate literatures and indicates that existential concerns play an important role in the propensity to engage in political violence, by increasing concerns regarding justice over the rational cost-benefit utility of such actions.

The appeal of justice over utility considerations for violence may seem to suggest that MS induces an irrational aggressiveness toward adversaries. The results of Study 1, however, indicate that such a conclusion would be an overstatement of the effect. Study 1 clearly demonstrates that MS induces violence only when the provocation is severe enough to justify retaliation, but when the initial provocation is inconsequential there is no significant effect for MS. Thus, retaliation following MS is not automatic and is dependent on the perception that an attack against one’s group bears severe consequences that call for retribution. Once it has been established that an attack was severe, the motivation for retaliation is based on a desire to deliver justice, and not on the likelihood that retaliation would reduce the risk of future attacks. These findings suggest that the process from existential threat to support for violent solutions to conflict involves both reason and passion such that only severe enough threats prompt retaliation, but once the desire for retaliation is activated it is no longer sensitive to practical considerations.

The research presented here echoes Becker’s (1975, p. 140) view that “man cannot feel right unless he lives the heroic victory over evil, the assurance of immortality.” Fighting for justice and vanquishing evil amplify the moral differences between *us* and *them* and bolsters the belief that conflict with another group is not just a selfish quarrel over material resources, but rather, an epic battle of good versus evil. In this state of mind, it is no wonder that practical considerations of costs and benefits carry little weight. These conclusions extend beyond TMT, and are in keeping with a variety of perspectives on political violence and existential threats, morality, deontological reasoning, and defensive reactions (e.g., McGregor et al., 2007; Ginges and Atran, 2011; Haidt, 2012).

The findings of the current research suggest that the rhetoric of in-group moral justification is a catalyst of intergroup violence, especially when existential threats are salient, as they often are in protracted violent conflicts. Absolutist conceptions of justice can be viewed as antithetical to peace because peace requires recognition of the claims of the other side, realistic assessment of possibilities, and acceptance of painful compromises that often involve relinquishing desires for vengeful justice. When people hold strong convictions about the justice of their group’s position, they perceive the conflict in zero-sum terms, are less likely to support compromise with adversaries, and are more likely to believe that violence is inevitable (Bar-Tal and Halperin, 2010).

It is important to emphasize that in this research we used a concrete economic definition of utility: the weighing of tangible benefits against the costs involved. Utility, however, can also be construed more broadly and abstractly. Recent evolutionary thinking suggests that violent retributions and concerns for justice may have served important pragmatic adaptive functions during evolutionary history (e.g., McCullough, 2008), such as deterring potential aggressors by sending a clear message that acts of aggression are not worth the risk. It is quite possible that the selective advantage of justice-related thinking over evolutionary history makes such responses attractive, even when they do not seem to have clear concrete benefits in a specific instance.

One should also note that Studies 1 and 2 differ in the conditions motivating violence. Whereas in Study 1 the motivation for violence is clear retribution that follows *lex talionis* – an eye for an eye, in Study 2 the term retribution may not accurately capture the motivation underlying support for violence. Rather, it is preemption of the possibility of violence from a dangerous adversary that is the instigator of aggression. These differences may explain the hesitation to support an attack on Iran in Study 1, compared with the lack thereof in Study 2.

It is also important to note that this research was conducted on a specific cultural, religious, and age group, young Israeli Jews, and in the context of an ongoing protracted conflict with the Palestinians, and on a potentially disastrous conflict with Iran. Although the psychological processes underlying intergroup conflicts share many similarities (Bar-Tal and Halperin, 2010), future research is needed to establish that the findings of the current research pertain to other conflicts as well. Of particular interest is whether justice motivations serve as a catalyst for violence in other conflicts and other regions. This research contributes to a growing understanding that support for violence in intergroup conflicts is indeed rooted in the implications of such action for one’s sense of meaning and justice than in the utility of such actions for one’s improving one’s concrete circumstances.

This research indicates that when existential issues are at stake there is danger that conflict will stray from a normative, rational process wherein costs and benefits are carefully considered, and will revert to an epic battle of good over evil that often defies logic and places the lives of millions at peril, even when the chances of success are low. The results of the current research shed experimental light on real-life eruptions of violent conflict and provide a new avenue for understanding the processes that lead to the preference for violent solutions to political problems – even when these solutions make little practical sense.

## Author Contributions

GH initiated this research, planned and ran the studies, and led the writing of the manuscript.

## Conflict of Interest Statement

The authors declare that the research was conducted in the absence of any commercial or financial relationships that could be construed as a potential conflict of interest.
